# Neighborhood clustering analysis to define epithelial–stromal interface for tumor infiltrating lymphocyte evaluation

**DOI:** 10.1016/j.jpi.2025.100465

**Published:** 2025-08-06

**Authors:** Tony Yeung, Yi Zhang, Qianghua Zhou, Richard Burack

**Affiliations:** a Pathology and Laboratory Medicine, University of Rochester Medical Center, 601 Elmwood Avenue, Rochester, NY 14642, USA; b Department of Laboratory Medicine and Pathobiology, University of Toronto, 1 King's College Circle, 6^th^ Floor, Toronto M5S 1A8, ON, Canada

**Keywords:** Neighborhood clustering analysis, Epithelial–stromal interface, Tumor infiltrating lymphocytes, Spatial heterogeneity, Multiplex immunofluorescence imaging, CD8, T cell immunoglobulin and mucin-domain containing-3 (TIM3), Immune checkpoint blockade therapy, High-grade serous carcinoma, Low-grade endometrial carcinoma

## Abstract

Evaluation of tumor infiltrating lymphocytes as recommended by current guidelines is largely based on stromal regions within the tumor. In the context of epithelial malignancies, the epithelial region and the epithelial–stromal interface are not assessed, because of technical difficulties in manually discerning lymphocytes when admixed with epithelial tumor cells. The inability to quantify immune cells in epithelial-associated areas may negatively impact evaluation of patient response to immune checkpoint therapies. Innovative spatial analysis techniques have emerged that can directly address challenges associated with quantification of lymphocytes in specialized regions like the interface. In this study, we apply supervised neighborhood clustering analysis (via an open-source application CytoMAP) to assess the spatial distribution of CD8+ T cells, CD8+ TIM3+ (T cell immunoglobulin and mucin-domain containing-3) exhausted T cells, and TIM3+ CD8- macrophages on a gynecological tumor microarray. Neighborhood clustering analysis is adept at objectively mapping the epithelial–stromal interface alongside the epithelial and stromal region of each tumor under a three-compartment model. When tumors are partitioned by the conventional two-compartment model (epithelial and stromal region only), the highest density of total CD8+ T cells is found in the stromal region in a slight majority of tumors. In contrast, the interface region surpasses both the epithelial and stromal region in holding the highest density of CD8+ T cells when this unique region is incorporated into the three-compartment model. Further subset analysis shows higher proportion of CD8+ TIM3+ exhausted T cells within the interface and epithelial region, as compared to CD8+ TIM3- T cells which span from the stroma to the interface. These results highlight the utility of implementing quantitative spatial technique and immune subset analysis in the assessment of tumor infiltrating lymphocytes, and underscore the potential significance of the under-reported tumor epithelial–stromal interface.

## Introduction

Histopathological assessment of tumor infiltrating lymphocytes (TILs) is largely restricted to the stromal regions of tumor.^1^, ^2^, ^3^ There is an inability to reproducibly quantify TIL within intra-tumoral epithelial regions, thus leading to their omission in the TIL evaluation.^4^ Whereas specialized peri-tumoral locations have also been recognized (such as the 1-mm invasive margin at leading edge of tumor), they are not yet fully adapted into the routine TIL evaluation.^2^^,^^3^^,^^5^ The under-reporting of intra-tumoral epithelial regions and other specialized regions may negatively impact the clinical utility of TIL in guiding immune checkpoint therapy for some patients.

In this study, we illustrate the utility of neighborhood clustering analysis to define the tumor epithelial–stromal interface. In epithelial malignancies such as ovarian and endometrial carcinomas, there are sub-millimeter-thick intersecting or infiltrating stroma in between epithelial tumor deposits, where epithelial tumor cells are in close proximity to the intersecting stromal cells. Under the current practice, this specialized area (interface) is not distinguished either from the deeper/inner epithelial region or the broader peri-tumoral stromal region, but may capture unique tumor–immune interactions. Whereas machine learning tools can be applied to detect and quantify the abundance of lymphocytes,^6^, ^7^, ^8^, ^9^ there remains a lack of consensus spatial framework with which to properly allocate the distribution of infiltrating lymphocytes across the entirety of each tumor. In this regard, Stoltzfus et al. provided an open-source and user-friendly neighborhood clustering analysis tool (CytoMAP), where distinct neighborhoods (including the epithelial–stromal interface) can be defined based on localized composition of tumor cells, stromal cells, and immune cells.^10^

In our workflow, a duplex immunofluorescence staining panel consisting of CD8 and T-cell immunoglobulin and mucin-domain containing-3 (TIM3) was used to assess CD8+ TIM3- T cells, CD8+ TIM3+ exhausted T cells,^11^ and TIM3+ (presumed) macrophages on a tumor microarray (TMA) of high-grade serous carcinomas (HGSCs) and low-grade endometrial carcinomas. Using CytoMAP, we generated visually intuitive spatial map that highlights distinct neighborhoods with unique mix of T cells, macrophages, tumor epithelial cells, and stromal cells. When the supervised neighborhood clustering analysis is simplified to a three-compartment model, each tumor is readily partitioned into tumor epithelial region, stromal region, and the tumor–stromal interface. By delineating the interface, we observe highest density of total CD8+ T cells and TIM3+ macrophages in this unique region. Further subset analysis shows differential spatial distribution, where CD8+ TIM3- T cells span between the stromal region and the interface, as compared to CD8+ TIM3+ exhausted T cells that distribute between the interface and epithelial region. In summary, we demonstrate a robust and accessible approach to assess intra-tumoral regional heterogeneity in TILs. The tumor epithelial–stromal interface may reveal critical tumor-immune dynamics that are relevant for understanding patient response to immune checkpoint therapies.^1^^,^^12^

## Methods and materials

All cases and clinical data were from University of Rochester Medical Center (URMC); the protocol was approved and the need for informed consent was waived by the Research Subjects Review Board.

### Construction of a gynecological tumor tissue microarray for optimization of immunofluorescence staining

A gynecological tumor tissue microarray was constructed with 10 HGSCs and 5 low-grade (FIGO Grade 1) endometrial carcinomas. Cases were chosen randomly from 2020 and 2021 from the URMC pathology department archive. Four random regions of tonsil lymphoid tissue were also included as fiducial marker (at the upper left corner of the TMA).

### Immunofluorescence staining of gynecological tumor microarray

TMA slide was processed for immunofluorescence staining according to previous protocol^13^^,^^14^ and instructions from the tyramide superboost kit (ThermoFisher Scientific, Waltham, MA, USA, catalog #B40913). Briefly, slide went through dewaxing and rehydration at the clinical immunohistochemistry (IHC) lab, followed by antigen retrieval at pH 6 with citrate buffer (Agilent Dako, Santa Clara, CA, USA, catalog #S236984) for 30 min, peroxidase blocking (Leica Biosystems, Deer Park, IL, USA, catalog #RE7101-CE) for 10 min, and goat-serum (5%) blocking for 30 min. In cycle one, the tissue was incubated with mouse anti-TP53 antibody at 1:100 (clone DO-7; ThermoFisher Scientific/Invitrogen, Waltham, MA, USA, catalog #MA5-12557) for 2 h, followed by goat anti-mouse horseradish peroxidase (HRP) conjugated secondary antibody (ThermoFisher Scientific, Waltham, MA, USA, catalog #B40961) for 1 h and then tyramide-Alexa555 reaction (ThermoFisher Scientific, Waltham, MA, USA, catalog #B40955) for 7 min. In cycle two, the slide was first heated to strip off prior antibodies at pH 6 with citrate buffer (Bond epitope retrieval solution 1; Leica Biosystems, Deer Park, IL, USA, catalog # AR9961) for 30 min, followed by incubation with both rabbit anti-TIM3 antibody at 1:66 (clone D5D5R; Cell Signaling Technology, Danvers, MA, USA, catalog #45208S) and mouse anti-CD8 antibody (Cell Signaling Technology, Danvers, MA, USA, catalog #70306S) for 2 h. The slide was then incubated with goat anti-rabbit HRP conjugated secondary antibody (ThermoFisher Scientific, Waltham, MA, USA, catalog #B40962) and goat anti-mouse-Alexa647 secondary antibody at 1:100 (ThermoFisher Scientific, Waltham, MA, USA, catalog #A48289) for 1 h. Tyramide reaction was performed with tyramide-Alexa488 (ThermoFisher Scientific, Waltham, MA, USA, catalog #B40953) for 4 min. The nuclei were then stained with DAPI (ThermoFisher Scientific/Invitrogen, Waltham, MA, USA, catalog #D21490) at 4 μM for 20 min.

We compared the immunofluorescence staining pattern of TP53 from this protocol with the standard IHC protocol, and observed differences in staining pattern and quality. We found that pH 6 retrieval was suboptimal, whereas pH 9 retrieval was necessary for the TP53 antibody. Because the TMA slide was retrieved at pH 6, the TP53 staining (Alexa555) is not included for further analysis in this study.

### Whole-slide scanning

Whole-slide scanning of the gynecological TMA was performed using the Olympus VS110/VS120 scanner (Evident Corporation, Tokyo, Japan). The fluorescence staining was scanned at 20× objective under the fluorescence mode using four channels (DAPI, FITC, Texas Red, and CY5) and exposure setting similar to previous protocol.^13^ The H&E slide was scanned at 20× objective under bright field mode.

### Single-cell image analysis (cellular phenotyping) in QuPath

Nuclear segmentation was performed in QuPath 0.5.0,^15^ using the StarDist nuclear detection algorithm (dsb2018_heavy_augment.pb model for DAPI-stained nuclei) (https://qupath.readthedocs.io/en/stable/docs/deep/stardist.html).^16^

For the fluorescence image, cellular phenotyping was performed using the object classifier in QuPath to define six different cell types (CD8+ TIM3- T cell, CD8+ TIM3+ exhausted T cell, TIM3+ CD8- presumed macrophage, stromal cell, epithelial cell, and DAPI smudges). Representatives of each cell type (around 10–30 cells per cell type) were identified manually by drawing region of interest around these cells on the fluorescence image as training input of the object classifier. Cells with a range of fluorescence intensities (beyond background level) were included for the CD8 and TIM3 channel when training CD8+ TIM3- T cell, CD8+ TIM3+ T cell, and TIM3+ CD8- macrophage. After training, the object classifier provided the designation of each cell as one of the six cell types.

Nuclear artifactual objects that appear as DAPI smudges on the fluorescence image were trained to be recognized by the QuPath object classifier. By using object classifier to identify these artifactual objects, we can avoid the somewhat subjective and time-consuming process of manually deleting them in an ad hoc manner during visual review of image. We also retain these artifactual objects throughout the analysis, so that we can potentially identify certain TMA spots that may be affected by higher amount of these artifacts.

### Neighborhood clustering analysis

Supervised neighborhood clustering analysis was performed using the open-source application CytoMAP (Gerner Lab, University of Washington, Seattle, WA, USA) on the Matlab R2022b platform (MathWorks, Natick, MA, USA). The analysis workflow is described in Stoltzfus et al.^10^ Briefly, single-cell data for each cell type generated from QuPath are saved in its own csv file. The input files for the different cell types from the immunofluorescence image are imported into CytoMAP using its graphical user interface (GUI). Neighborhoods are generated based on the input cell types and a specified neighborhood radius. Neighborhoods are then clustered into user-specified number of regions. Spatial maps and region statistics are generated. Neighborhood region percentages are based on number of cells in a particular neighborhood region over total number of cells. Certain neighborhood region data are exported as csv files for downstream handling in FlowJo version 10.10.0 (BD Biosciences, Franklin Lakes, NJ, USA) or for importing back to QuPath for visual display (https://forum.image.sc/t/there-and-back-again-qupath-cytomap-cluster-analysis/43352). Additional details are provided within figure legends.

### Statistical analysis

Prism 9.1.0 (GraphPad Software, Boston, MA, USA) is used for plot generation and statistical analysis. Additional details are provided within the “Results” section.

## Results

### Cellular phenotyping by CD8, TIM3, and DAPI-nuclear features to define immune cell subsets, tumor, and stromal cells

**Fig. 1:**
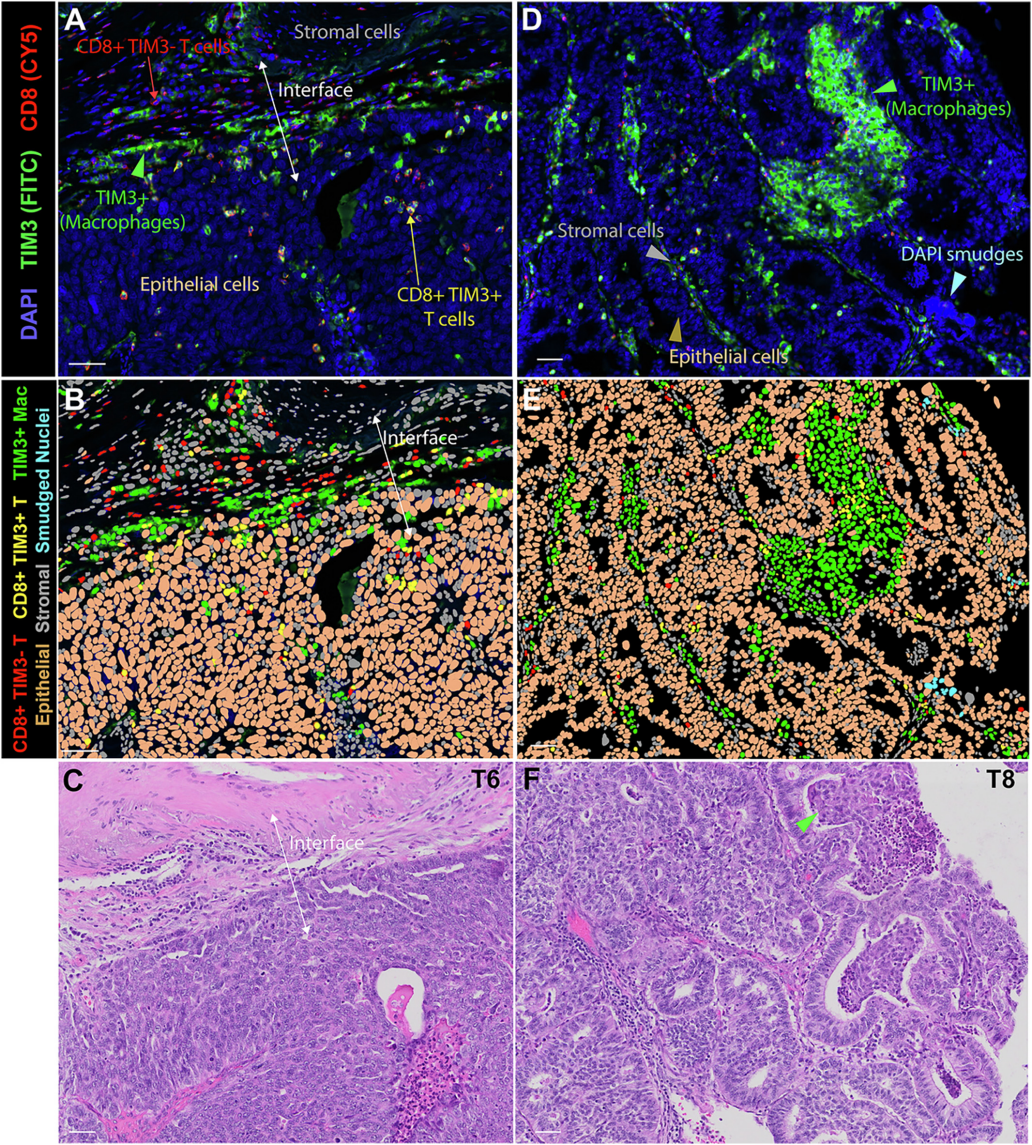
Cellular phenotyping by CD8, TIM3, and DAPI-nuclear features to define immune cell subsets, tumor and stromal cells. A, D. Portion of whole-slide fluorescence image of the gynecological tumor microarray showing high-grade serous carcinoma (case T6 in A) and low-grade endometrial carcinoma (case T8 in D) after staining with CD8 (red), TIM3 (green), and DAPI (blue). Representative CD8+ TIM3- T cells and CD8+ TIM3+ exhausted T cells are indicated by red and yellow arrows, respectively. (Higher magnification view of the area in A also shown in Fig. 5). Epithelial cells and stromal cells are indicated by the overlying beige and grey text label or arrowheads. TIM3+ CD8- presumed macrophages are indicated by green arrowhead. Bright DAPI nuclei that are smudged are indicated by the cyan arrowhead. B, E. Diagram of cellular phenotype where nuclear mask of each cell is color-coded based on one of six cellular phenotypes as determined by the QuPath object classifier as follows: CD8+ TIM3- T cell (red), CD8+ TIM3+ exhausted T cell (yellow), TIM3+ CD8- presumed macrophage (green), epithelial cell (beige), stromal cell (gray), and DAPI smudge (cyan). C, F. Hematoxylin-eosin (H&E) image of the respective area from another serial section of the gynecological tumor microarray. Lymphocytic infiltrate at tumor epithelial–stromal interface shown in C. Intra-luminal collections of macrophages are shown in F (green arrowhead). Tumor epithelial–stromal interface indicated by double-headed white arrow in A–C. Scale bar = 50 μm for all.

We applied a custom low-multiplex immunofluorescence staining protocol for CD8 and TIM3 on a gynecological TMA composed of 10 HGSC and 5 low-grade endometrial carcinomas. The inclusion of low-grade endometrial carcinomas provides contrast with HGSCs, such as density of epithelial deposits, pattern, and composition of intersecting/infiltrating stroma. Nuclear segmentation was performed on the whole-slide fluorescence image in QuPath to enable single-cell analysis. Cellular phenotyping was performed using object classifier in which six unique cell types were defined based on CD8 and TIM3 fluorescence characteristics, and DAPI-stained nuclear features (Fig. 1A–B, D–E; see “Methods”).^13^

Using case T6 (HGSC) as an example, the object classifier is able to distinguish epithelial (primarily tumor) cells (Fig. 1A, B beige) from stromal cells using DAPI-nuclear features, with acceptable performance (by comparing to the H&E image, Fig. 1C); where stromal cells (Fig. 1A, B gray) are defined as remainder of cells in the stroma that are not CD8+ T cells or TIM3+ (presumed) macrophages. CD8+ TIM3- T cells span primarily between the broader stromal region and the epithelial–stromal interface (Fig. 1A–B in red). In contrast, CD8+ TIM3+ double-positive exhausted T cells are enriched at the epithelial–stromal interface and in the epithelial region (Fig. 1A–B in yellow). In case T8 (low-grade endometrial carcinoma), there is intersecting stromal and intra-luminal accumulation of TIM3+ CD8- (presumed) macrophages (Fig. 1D–E in green). Their presumed identity as macrophages is corroborated by the cytomorphological appearance of these cells on the H&E image (Fig. 1F green arrowhead). Representative area of each case is shown in magnified view in Fig. S1.

### Spatial plot of tumor epithelial cell to CD8+ T cell distance to highlight hot and cold tumor regions

**Fig. 2:**
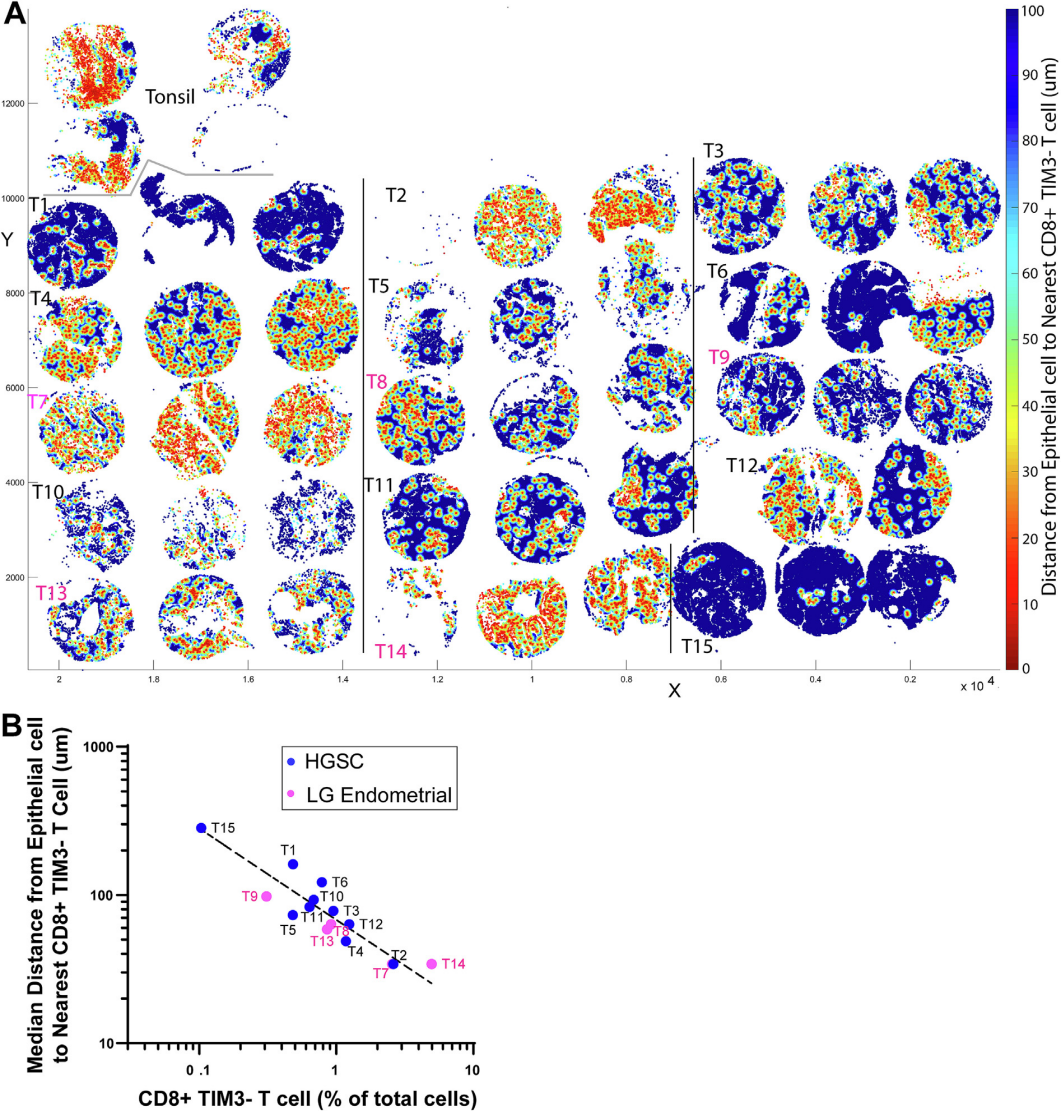
Spatial plot of tumor epithelial cell to CD8+ TIM3- T cell distance to highlight hot and cold tumor regions. A. x–y scatterplot showing only the epithelial cells of each tumor, where each cell is color-coded based on the distance to its nearest CD8+ TIM3- T cell (heat scale shown on far right). x-axis and y-axis in μm. Cases are color-coded according to legend in B below. B. CD8+ TIM3- T cell (as percentage of total cells) plotted against median distance (in um) from each epithelial cell to its nearest CD8+ TIM3- T cell. Cases are labeled by their number and color-coded according to the legend. Best-fit line on the log-log plot shown.

As a commonly used spatial analysis tool, distance measurement provides informative spatial relationship between two different cell types. By plotting the distance between every tumor epithelial cell and its nearest CD8+ TIM3- T cell neighbor, we see *cold tumor regions* in case T1, T6, T15, where CD8+ TIM3- T cells are 100+ μm away from most of the tumor cells (Fig. 2A in blue). In contrast, *hot tumor regions* in case T2, T7, T14 show tumor epithelial cells in much closer proximity to CD8+ TIM3- T cells (Fig. 2A in red).

At the bulk population level, CD8+ TIM3- T cell abundance (as percentage of total cells) varies up to one-log level within either HGSCs or low-grade endometrial carcinomas, and trends inversely with the median distance between an epithelial cell and its nearest neighboring CD8+ TIM3- T cell (*r*^2^ = 0.85 for the best-fit line) (Fig. 2B).

### Neighborhood clustering highlights distinct neighborhoods with unique mix of different cell types

**Fig. 3:**
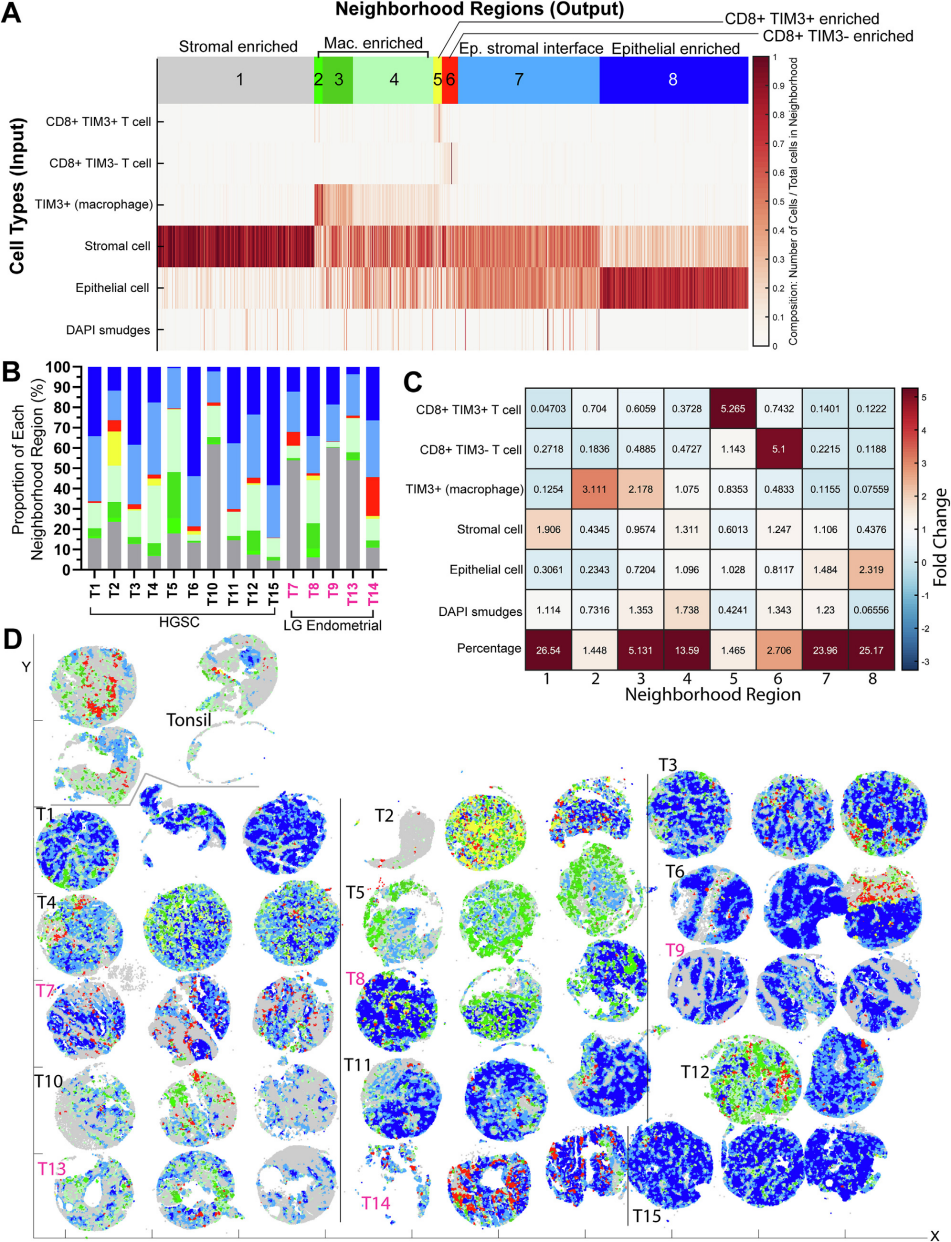
Neighborhood clustering highlights distinct neighborhoods with unique mix of different cell types. A. Supervised neighborhood clustering with six different cell types as input (left) and a neighborhood radius of 30 μm. Increased weighting is given to help accentuate clustering of low abundant cell types (weight of 8 for CD8+ TIM3- T cells and CD8+ TIM3+ T cells, weight of 5 for TIM3+ CD8- macrophages). Eight neighborhood regions are generated (numbered on top), each with a unique mix of the different cell types. B. Bar plot showing percentage of each neighborhood region for each case (see A for color code of each neighborhood region). C. Neighborhood statistics showing fold change of each cell type between different neighborhood regions. D. x–y scatterplot showing individual cell with color coding based on its association with a particular neighborhood region (see A for color code of each neighborhood region). Each grid mark on the x–y axis = 2 mm.

Higher-order spatial relationships can be demonstrated by neighborhood clustering analysis where distinct neighborhood regions are defined across the entirety of each tumor based on unique mix of different cell types that reside within a certain neighborhood radius.^10^ We performed supervised neighborhood clustering with six cell types as inputs (listed on the left side of Fig. 3A), and found eight neighborhood regions (from trial of 1 to 15 neighborhood regions) (listed on top of Fig. 3A) to best illustrate low abundant cell subsets (i.e., CD8+ T cells) within their micro-environments. Regions 1 and 8 are enriched in stromal cells or tumor epithelial cells, respectively (Fig. 3A–C). Region 7 captures the tumor–stroma interface (Fig. 3A–C). Regions 2–4, 5, 6 show enrichment in either TIM3+ macrophages, CD8+ TIM3+ exhausted T cells, or CD8+ TIM3- T cells, respectively (Fig. 3A–C). In Fig. 3D, the neighborhood map serves as a visually intuitive aid to enable rapid overview of unique immune regions within each tumor.

Case T2 is notable for Region 5 (in yellow) that is enriched in CD8+ TIM3+ exhausted T cells (Fig. 3B, D). Case T5 shows increased intra- and peri-tumoral regions enriched with TIM3+ macrophages (Regions 2–4 in different shades of green) (Fig. 3B, D). Case T6 shows peri-tumoral stroma enriched in CD8+ TIM3- T cells (Region 6 in red; TMA spot on right), whereas the intra-tumoral epithelial region is largely devoid of these T cells (Fig. 3B, D). This is contrasted by Case T14 (low-grade endometrial carcinoma) where CD8+ TIM3- T cells within Region 6 (in red) distribute in close proximity to the tumor epithelium (Fig. 3B, D**)**.

### Partition of tumor into three compartments for CD8+ T cell quantification

To expand quantification of TIL beyond the stromal region as currently performed,^2^, ^3^, ^4^, ^5^^,^^12^ we propose a three-compartment model that delineates the stromal region, epithelial region, and the epithelial–stromal interface. For typical translational studies of immune therapy response, this approach enables the evaluation of regional immune heterogeneity across these well-recognized regions.

As epithelial cells and stromal cells are the two most abundant cell types on our TMA, the neighborhood clustering analysis can readily render an epithelial region and a stromal region in a conventional two-compartment model, based on local enrichment of either of these two cell types. But by partitioning each tumor into three neighborhood regions, the clustering analysis identifies transitional areas in between epithelial region and stromal region that hold about half the abundance of both epithelial and stromal cells (Fig. S2A,C). This intermediate region essentially represents the epithelial–stromal interface for most cases (Fig. S2B,D; S1 top right panel of each case).

### Epithelial–stromal interface as a distinct region that holds the highest density of CD8+ T cells

**Fig. 4:**
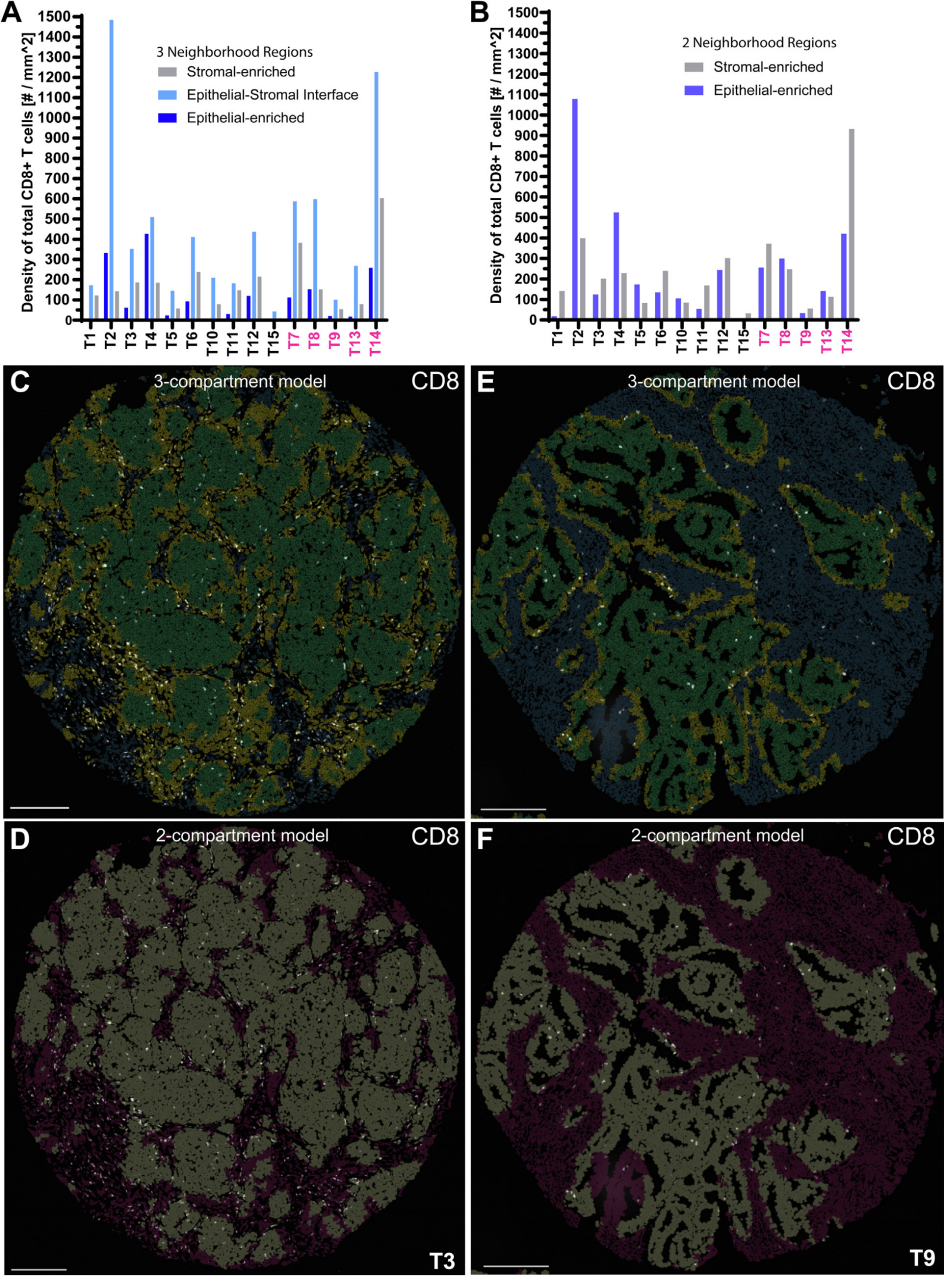

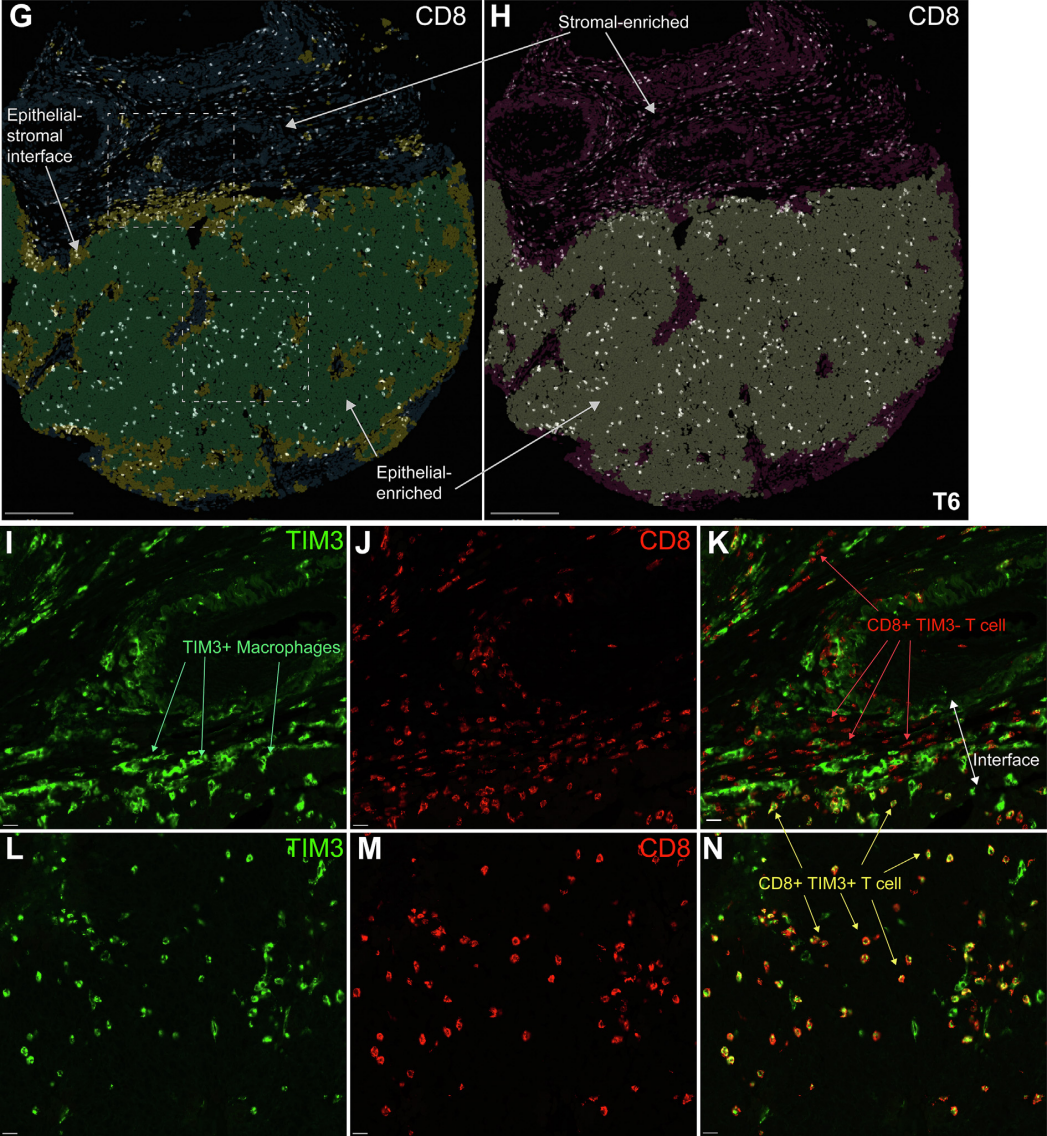
Epithelial–stromal interface as a distinct region that holds the highest density of CD8+ T cells. A–B. Bar plot showing density of total CD8+ (TIM3+ and TIM3-) T cells in the indicated neighborhood region under the (A) three-compartment model or (B) two-compartment model. (C–H) Overlay of fluorescence signal of total CD8+ T cells (TIM3+ and TIM3-) in white on top of neighborhood regions under the (C, E,G) three-compartment model (stromal region in blue-gray, interface in tan-yellow, epithelial region in cyan-green), or (D, F, H) two-compartment model (stromal region in magenta, epithelial region in gray). (I–N) Fluorescence image showing (I, L) TIM3, (J, M) CD8, and (K, N) merge of both. I–K, L–N are magnified view of top and bottom dashed box in G, respectively. Case label included in the bottom right corner of D, F, H. Scale bar = 250 μm in C–H; 20 μm in I–N.

In this simplified three-compartment model, the epithelial–stromal interface holds the highest density of total CD8+ T cells in all cases (Fig. 4A). This contrasts with a slight majority of cases showing highest density of total CD8+ T cells in the stromal region under the conventional two-compartment model (epithelial region and stromal region) (Fig. 4B). The relative enrichment of total CD8+ T cells at the interface can still be appreciated even for moderately immune-poor tumors (i.e., cases T3, T9) (Fig. 4C,E). In contrast, this unique pattern of T cell distribution may more likely be overlooked using the two-compartment model (Fig. 4D,F). These results are also contrasted with the current gold-standard TIL evaluation (manual TIL assessment), where only the stromal TIL coverage is reported (Fig. S3A).

Additional subset analysis shows relative preference for epithelial region over stromal region for CD8+ TIM3+ exhausted T cells (Fig. S3B,C). The epithelial region-to-stromal region ratio of cell density is higher for CD8+ TIM3+ exhausted T cells than CD8+ TIM3- T cells in 13 of 15 cases, indicating relatively more CD8+ TIM3+ exhausted T cells in the epithelial region (Fig. S3D). Conversely, CD8+ TIM3- T cells are relatively more abundant in the stromal region (Fig. S3D–F). Case T6 is a prototypical example (Fig. 4G,H) in which CD8+ TIM3- T cells largely reside in the stroma and interface regions (Fig. 4I–K), whereas CD8+ TIM3+ exhausted T cells predominate in the interface and epithelial regions (Fig. 4L–N).

## Discussion

This study illustrates how the use of immunofluorescence staining and innovative spatial analysis tool helps overcome major challenges with the current quantification of TIL.^2^, ^3^, ^4^, ^5^ By providing a simplified three-compartment model to report regional heterogeneity in tumor infiltrating T cell subsets and macrophages, the significance of their spatial distribution may be more meaningfully evaluated in studies evaluating response to immune checkpoint therapies.^1^^,^^17^ Our results show that the tumor epithelial–stromal interface holds the highest density of CD8+ T cells (Fig. 4A) and TIM3+ macrophages (Fig. S3G,H), thus providing rationale for assessing this specialized region. In contrast, the epithelial region is relatively devoid of these immune cells in many but not all of the cases (Figs. 4A; S3D), and this epithelial-region immune paucity is not predicted based on the manual stromal TIL assessment (i.e., comparing case T1 and T2 in Figs. 4A; S3A).

The growing demand for spatial analysis in pathology necessitates further development and validation of spatial analysis framework and software platform. In this regard, whereas our workflow is reasonably accessible (based on available open-source applications that have GUI for enhanced usability^10^^,^^15^), it is important to further standardize and validate these tools before they can be implemented for regulatory or diagnostic use. The accuracy of our supervised approach depends on the ability to correctly identify various cell types on the immunofluorescence image, and then properly partition tumor into epithelial and stromal regions and the epithelial–stromal interface. Whereas we derive generally acceptable performance from the QuPath object classifier in phenotyping the six different cell types, we recognize occasional inaccuracies in the discrimination between epithelial cells, immune and stromal cells (Fig. S1E, S1K red arrow), leading to under- or over-representation of particular neighborhood region (Fig. S4). Future improvement to address this issue may be through applying epithelial and pan-immune markers (i.e., EpCAM, PAX8, and CD45) to help further distinguish between these cell types. For the spatial partitioning framework, the clinical utility of the tumor epithelial–stromal interface can be further validated in subsequent study using the neighborhood clustering approach as outlined in this study.

## CRediT authorship contribution statement

TY and RB contributed to research design. RB provided research mentorship. TY and YZ optimized the low-multiplex immunofluorescence staining protocol. TY performed the immunofluorescence staining of the gynecological tumor microarray, cellular phenotyping of single-cell data in QuPath, spatial analysis in CytoMAP, manual stromal TIL evaluation, manual assessment of T cell distribution, and statistical analysis. TY and RB wrote the manuscript. YZ and QZ provided critical evaluation of spatial analysis results. All authors helped with revision of manuscript and approved the manuscript.

## Declaration of competing interest

The authors declare that they have no known competing financial interests or personal relationships that could have appeared to influence the work reported in this article.

## Data Availability

Microscopy images will be accessible in the public repository Biostudies (https://www.ebi.ac.uk/biostudies/bioimages/studies/S-BIAD1703).

## Appendix A Supplementary data

Figure S1 Magnified view of representative area of each tumor. (**A**–**O**) Representative area of each tumor is shown, with their case label and tumor type indicated on top. **Top left**: Fluorescence image showing DAPI-stained nuclei (blue), CD8 (red), TIM3 (green). **Bottom left**: Diagram of cellular phenotype where nuclear mask of each cell is colored based on cell type assigned by QuPath object classifier (see legend for color coding). **Top right**: x-y scatter plot showing each cell color coded based on its association with one of three neighborhood regions. (see legend for color coding). x-y axis in um. **Bottom right**: H&E image of the representative area from another serial section. Red arrows in **E**, **K** point to inaccuracies by the QuPath object classifier that mis-identified epithelial cells as stromal cells, which in turn resulted in over-representation of epithelial-stromal interface or stromal region, respectively. Figure S1

Figure S2 Partition of each tumor into three compartments for CD8+ T cell quantification. **A.** Supervised neighborhood clustering with six different cell types as input (left) and a neighborhood radius of 30um. Three neighborhood regions are generated (numbered on top), each with a unique mix of the different cell types. **B**. Bar plot showing percentage of each neighborhood region for each case (see **A** for color code of each neighborhood region). **C**. Neighborhood statistics showing fold change of each cell type between different neighborhood regions. **D**. x-y scatterplot showing individual cell with color coding based on its association with a particular neighborhood region (see **A** for color code of each neighborhood region). Cases are labeled by their number where red color denotes low-grade endometrial carcinoma and black color denotes high-grade serous carcinoma. x-y axis in um. Figure S2

Figure S3 Density of CD8+ T cell subsets and TIM3+ macrophages in the specified neighborhood regions. (**A**) Manual assessment of tumor infiltrating lymphocytes (TIL) in the stromal regions based on current guideline. Quantification is provided by a range of 5 % or 10 %. (**D**) Epithelial region-to-stromal region ratio of T cell or macrophage density. Bar plot showing density of (**B**,**C**) CD8+ TIM3+ T cells, (**E**,**F**) CD8+ TIM3- T cells, (**G**,**H**) TIM3+ CD8- macrophages in the indicated neighborhood region, under the (**B**,**E**,**G**) 3-compartment model or (**C**,**F**,**H**) 2-compartment model. Cases are labeled by their number and color coded where magenta denotes low-grade endometrial carcinoma, remainders are high grade serous carcinoma. Figure S3

Figure S4 Comparison of manual vs. digital assessment of CD8+ T cell distribution. Manual assessment of total CD8+ T cells (both TIM3+ and TIM3-) was performed among the three compartments of each tumor (tumor epithelium, stroma, interface), and results were compared with the digital approach (using QuPath and CytoMAP). In the manual assessment by pathologist (TY), the interface was defined as an approximately 40-um or 4-cell-diameter space between tumor epithelium and stroma. Only 1 of 3 cores was assessed manually for T2, T4, T7, T8, T14, while all cores were assessed for remaining cases. The percentage of total CD8+ T cells in each tumor compartment was shown in the bar plot. Case T5 showed the most difference between manual and digital approach, for the quantification of CD8+ T cells in the tumor epithelium vs. interface region. This was due to inaccurate cell phenotyping by the QuPath object classifier that mis-identified epithelial cells as stromal cells. Figure S4
